# Adalimumab-Induced Lupus Serositis: A Case Report and Review of the Literature

**DOI:** 10.7759/cureus.34568

**Published:** 2023-02-02

**Authors:** Sandy Lee, Anna Lafian, Tandis Mahani, Mehrnaz Hojjati

**Affiliations:** 1 Department of Rheumatology, Keck School of Medicine of the University of Southern California, Los Angeles, USA; 2 Division of Rheumatology, Department of Internal Medicine, Loma Linda University School of Medicine, Loma Linda, USA; 3 Department of Internal Medicine, University of California, Riverside, USA

**Keywords:** drug-induced lupus, tnf-alpha antagonist, pericardial effusion, arthritis, adalimumab

## Abstract

Tumor necrosis factor-alpha (TNF-alpha) antagonist use is prevalent for the treatment of autoimmune diseases, including psoriasis, ankylosing spondylitis, and rheumatoid arthritis. Since the onset of its use over the last couple of decades, there have been increasing reports of drug-induced antibodies and antitumor necrosis factor-alpha-induced lupus (ATIL). Herein, we present a case of pericarditis induced by tumor necrosis factor-alpha antagonist, adalimumab. A 61-year-old male with psoriatic arthritis treated with adalimumab injections for five years presented with dyspnea, chest tightness, and three-pillow orthopnea. Echocardiogram showed moderate pericardial effusion with early signs of tamponade. Adalimumab was discontinued. He was started on colchicine and steroids for a high suspicion of drug-induced serositis. With the increased use of tumor necrosis factor-alpha antagonists, adverse reactions such as ATIL will become more common. Such cases need to be reported to spread awareness of this possible complication and avoid any delay in treatment and care.

## Introduction

The development of tumor necrosis factor-alpha (TNF-alpha) antagonists has greatly influenced the treatment of rheumatologic diseases such as plaque psoriasis, ankylosing spondylitis, and rheumatoid arthritis when first-line therapies fail. Etanercept, infliximab, adalimumab, certolizumab, and golimumab are the five TNF-alpha inhibitors currently on the market, with the first three listed most commonly prescribed. Biologics, as these agents are also called, are monoclonal antibodies, except etanercept, which is a fusion protein of the p75 fragment of the TNF-alpha receptor and the Fc component of the human immunoglobulin G1 [1-3]. Side effect profiling of these biologics is relatively safe with a black box warning of fatal complications of diseases such as tuberculosis and pneumocystis pneumonia that warrants investigation for dormant disease prior to starting the medication [3,4]. A post-marketing surveillance of adalimumab, after its release in 2002, deemed the drug relatively safe and well tolerated with benefits outweighing risks in those receiving this biologic for the control of their rheumatoid arthritis [4].

However, given the nature of TNF-alpha inhibitors in altering immunogenicity, antidrug antibodies are commonly detected in serial blood studies with some rare patients continuing on to develop lupus or lupus-like syndromes [1,5-7]. Antitumor necrosis factor-alpha-induced lupus (ATIL) should be considered if there is at least one serological and one clinical feature of the American College of Rheumatology (ACR) classification criteria for lupus have been met. Few cases of drug-induced lupus (DIL) secondary to adalimumab have been reported, in contrast to the many cases reported with the use of etanercept or infliximab, as these drugs have been in use for a longer period of time [1,5,7,8]. We present a case of adalimumab-induced pericarditis.

## Case presentation

A 61-year-old male with a medical history of hypothyroidism and psoriatic arthritis presented to our medical center with the complaint of dyspnea, three-pillow orthopnea, and substernal chest pain for six weeks. Symptoms were exacerbated by exertion and improved with rest. He denied any recent upper respiratory tract infections or gastrointestinal infections. He was given a trial of furosemide by his outpatient physician; however, the medication did not alleviate his symptoms. He denied any cardiac history. Scheduled home medications included adalimumab, metoprolol tartrate, and valsartan-hydrochlorothiazide. His adalimumab injections were scheduled bimonthly, and he had been on the medication for five years with the most recent dose administered three weeks prior to presentation. Family history was only notable for coronary artery disease. His social history was positive for a prior 21-pack-year tobacco use, which he quit in 2006. He previously worked as a plumber.

The patient was hemodynamically stable. His physical examination was significant for tachycardia, jugular venous distention, and skin findings of scaly plaques on all extensor surfaces and his torso with the largest plaque measuring 3 cm.

Upon presentation, cardiac enzymes were negative. Bedside ultrasound showed a normal left ventricular ejection fraction and a large-sized pericardial effusion. He had an elevated white blood cell count of 15,000 on admission. Hemoglobin and hematocrit were normal and stable throughout his stay. He also had asymptomatic thrombocytosis with a platelet count averaging 500 billion/L. His respiratory viral panel was positive for coronavirus strain 229E. Inflammatory markers C-reactive protein and erythrocyte sedimentation rate were elevated.

Interestingly, the antinuclear antibody human epithelial 2 (ANA-HEp-2) substrate came back highly positive with a titer of 1:2560 in a homogenous pattern. The antinuclear antibody panel was positive for elevated anti-Sjögren’s syndrome-related antigen A autoantibodies (anti-SSA/Ro autoantibodies) of greater than 8 AI (reference range: 0.2-0.9 AI), anti-Smith antibody positive at greater than 8 AI (reference range: 0-0.9 AI), and a chromatin antibody positive at 1.2 AI (reference range: 0.2-0.9 AI). Anti-histone antibodies were also positive at 2.8 U (reference range: <1 U). Complement proteins were within the normal range.

He was evaluated by cardiology and started on colchicine 0.6 mg twice a day and indomethacin 25 mg three times a day. His pericardial effusion was monitored with daily bedside ultrasound. After rheumatology evaluation, based on his workup, a diagnosis of new-onset drug-induced lupus was made, and the patient was started on pulse dose steroids with solumedrol 500 mg for three days with notable improvement of his shortness of breath. His bedside ultrasound showed dramatic improvement in pericardial effusion two days after treatment initiation. Pericardiocentesis was initially planned; however, given that the effusion significantly diminished, the procedure was canceled. The patient was subsequently transitioned to prednisone 60 mg and discharged on that dose along with colchicine. Adalimumab was discontinued.

He was seen at his outpatient follow-up appointment, and his prednisone was gradually tapered off. He remained asymptomatic with no recurrence of the pericardial effusion three months after his hospital discharge.

## Discussion

Antitumor necrosis factor-alpha-induced lupus (ATIL), a subset of drug-induced lupus (DIL), is a growing concern among healthcare providers as more of these medications are being prescribed. Many sources report an incidence of ATIL secondary to infliximab at 0.19%-0.22%, etanercept at 0.18%, and adalimumab at a mere 0.10% [4,6,7]. Since that time, there has been a rise in cases of ATIL reported. According to the updated BIOGEAS registry, DIL is the most common autoimmune disease associated with tumor necrosis factor-alpha (TNF-alpha) antagonists with 140 reported cases in the registry in 2009 with 25% being secondary to adalimumab use, 37% being secondary to infliximab use, and 33% being secondary to etanercept use.

However, despite the growing number of reported cases, there are no standardized criteria used to diagnose ATIL. When these biologics were released, the risk classification system of traditional DIL was not extended to the TNF-alpha inhibitors because of the difficulty of discerning ATIL from latent autoimmunity as many patients being treated may have already had predisposed autoimmune disease [1]. Nonetheless, criteria have been proposed to identify cases of ATIL, which include a positive set of serological markers such as positive antinuclear antibodies or double-stranded DNA, non-serological symptoms such as arthritis or serositis, and a temporal correlation between the symptoms and medication. However, because of a lack of conventional criteria in place, not all cases of reported ATIL follow the same criteria for diagnosis [1,5,7].

Reported cases of ATIL oftentimes do not meet at least four criteria of the 11, as outlined in the American College of Rheumatology guidelines for systemic lupus erythematosus (SLE) [6,9,10]. The BIOGEAS registry reported that the SLE criteria were only detailed in 97 of the 140 cases, of which only 40% of the cases met four or more criteria with a majority of 60% fulfilling three or fewer criteria. They even defined lupus-like disease as only needing to fulfill three criteria rather than four of the 11 [9,10].

Reported symptoms of ATIL are often the usual cutaneous features (malar rash and photosensitivity), arthritis, and serositis, but milder in comparison to idiopathic SLE [1,2,5,6,10]. Many reported ATIL cases had a high frequency of certain features such as high titers of antinuclear antibodies (79%) and anti-double-stranded DNA (72%), as well as cutaneous manifestations (67%), while others such as serositis (10%) were reported less frequently [2]. Anti-histone antibodies were only found in 17%-57% of ATIL cases, whereas they can be found in 95% of DIL cases, although fairly nonspecific given that these antibodies are also present in those with SLE or rheumatoid arthritis [5,11]. In addition, autoimmune induction by TNF-alpha inhibitors has other various presentations besides ATIL, including vasculitis and interstitial lung disease [2,9].

Our patient exhibited serositis and had high titer positive antinuclear antibodies, positive anti-histone antibody, and positive anti-Smith antibody, with a score of 11 based on the 2019 European Alliance of Associations for Rheumatology (EULAR)/American College of Rheumatology (ACR) classification criteria, meeting the classification criteria for the diagnosis of lupus, and our patient was on a TNF-alpha inhibitor. We also considered other differentials such as idiopathic pericardial effusion, drug sensitivity, and Dressler syndrome. Nevertheless, our suspicion of ATIL was solidified when his anti-histone antibodies resulted positive. However, one could argue that he may have had underlying lupus that was unmasked by the TNF-alpha inhibitor. The* *analysis of Ramos-Casals et al. [2] highlights the importance of serological screening before starting TNF-alpha inhibitors to ensure the absence of underlying positive autoantibodies. If preexisting autoimmunity does exist, the paper advises caution on the use of these biologics, especially in those with interstitial pulmonary disorders. Unfortunately, our patient did not have baseline serology done prior to starting adalimumab.

According to Ramos-Casals​​​​​​​ et al. [2]​​​​​​​, the treatment for ATIL largely depends on the severity of the disease. If ATIL is confined to only cutaneous or serological features, then it is safe to simply terminate the drug; however, if there is further organ involvement such as pulmonary or renal involvement, then immunosuppressive agents should be added to avoid further complications [5]. Our patient developed serositis in the form of pericardial effusion, and pulse dose steroids were initiated promptly to prevent life-threatening complications.

Because of the development of ATIL, our patient stopped taking adalimumab, despite good symptomatic control of his psoriatic arthritis with the medication. There is no evidence on the safety of re-challenging patients on alternative TNF-alpha inhibitors after ATIL development. Wetter and Davis [12] reported re-trialing five patients on an alternative TNF-alpha inhibitor despite their history of ATIL secondary to infliximab. Four of the five tolerated the alternate TNF-alpha inhibitor (three were switched to adalimumab, and the other was started on etanercept) without the induction of ATIL. Ten cases in total of re-challenging patients on TNF-alpha inhibitors have been found with the majority of the patients tolerating the alternative therapy. However, these studies were inconclusive as to which biologic is the better alternative. In addition, we should be mindful of the small sample sizes reported [8]. Nevertheless, these cases give way to the possibility of using alternative TNF-alpha inhibitors when all else fails.

## Conclusions

Given the increasing use of tumor necrosis factor-alpha (TNF-alpha) inhibitors, we will be seeing more cases of adverse reactions such as antitumor necrosis factor-alpha-induced lupus (ATIL). A similar case as ours was found in the literature review. This patient also developed pericardial effusion that was diagnosed in time and managed appropriately. As such, more cases of ATIL need to be reported to shed light on this syndrome and the significance of early diagnosis and appropriate treatment. Likewise, standardized criteria for ATIL need to be in place to diagnose appropriately and avoid the exclusion of ATIL cases when American College of Rheumatology guidelines for systemic lupus erythematosus criteria are not met. Preventative strategies, such as baseline serology before TNF-alpha antagonist initiation, should be established to distinguish idiopathic lupus from a reaction like ATIL. The current research is hopeful, but more investigation needs to be done before we completely understand the full spectrum of effects of the use of TNF-alpha inhibitors.
